# Analysis of the antioxidative function of the radioprotective Japanese traditional (Kampo) medicine, hangeshashinto, in an aqueous phase

**DOI:** 10.1093/jrr/rrv023

**Published:** 2015-04-16

**Authors:** Chinami Matsumoto, Emiko Sekine-Suzuki, Minako Nyui, Megumi Ueno, Ikuo Nakanishi, Yuji Omiya, Masato Fukutake, Yoshio Kase, Ken-ichiro Matsumoto

**Affiliations:** 1 Radio-Redox-Response Research Team, Advanced Particle Radiation Biology Research Program, Research Center for Charged Particle Therapy, National Institute of Radiological Sciences, 4–9–1 Anagawa, Inage-ku, Chiba-shi, Chiba 263–8555, Japan; 2 Tsumura Research Laboratories, Tsumura & Co., 3586 Yoshiwara, Ami-machi, Inashiki-gun, Ibaraki 300–1192, Japan; 3 Research Program for the Application of Heavy Ions in Medical Sciences, Research Center for Charged Particle Therapy, National Institute of Radiological Sciences, 4–9–1 Anagawa, Inage-ku, Chiba-shi, Chiba 263–8555, Japan

**Keywords:** hangeshashinto (HST), oral mucositis (OM), electron paramagnetic resonance (EPR), radiation; reactive oxygen species

## Abstract

Oral mucositis (OM) is a common and painful complication of radiotherapy for head and neck cancer. Hangeshashinto (HST), a Japanese traditional medicine, is known to alleviate radiotherapy- and/or chemotherapy-induced OM; however, the detailed mechanism has not yet been clarified. The aim of the present study was to clarify the details of the antioxidative functions of HST against reactive oxygen species (ROS) produced by radiation. The hydroxyl radical (•OH)–scavenging ability and the reduction ability was simultaneously measured using a modified electron paramagnetic resonance (EPR) spin-trapping method. The superoxide (O_2_^•−^)–scavenging ability was estimated by an EPR redox probing method. Water suspensions of powdered HST and of its seven constitutive crude drugs were tested. In addition, some of the main water-soluble ingredients of the crude drugs were also tested. HST was found to scavenge both •OH and O_2_^•−^. Furthermore, HST was observed to reduce relatively stable nitroxyl radicals. Glycyrrhizae Radix (kanzo), Ginseng Radix (ninjin), Zizyphi Fructus (taiso) and glycyrrhizin (an ingredient of kanzo) were all found to be relatively good •OH scavengers. Scutellariae Radix (ogon) and Coptidis Rhizoma (oren) demonstrated reducing ability. In addition, acteoside and berberine chloride, which are water-soluble ingredients of ogon and oren, respectively, also demonstrated reducing ability. Oren exhibited oxidative ability at higher concentrations, which may have a function in maintaining catalytic redox action. The antioxidative function of HST probably worked via a balance of scavenging ROS, reducing stable free radicals, and some minor oxidizing activities.

## INTRODUCTION

Oral mucositis (OM) is currently considered to be the crucial complication of cancer therapy, affecting 40–80% of patients undergoing chemotherapy and almost all patients undergoing radiotherapy of the head and neck region [1]. The consequences of OM are far-reaching and include the necessity for chemotherapy dose reductions, breaks in radiation treatment, cessation of cancer therapy, and, especially, a reduction in the quality of life of patients [2]. Lately, clinical guidelines [3–5] and several reviews have confirmed the necessity for increased emphasis on the management of OM [1]. Thus, prevention and treatment of OM is becoming recognized as highly significant in clinical practice.

Initiation of OM occurs immediately following the administration of radiation and/or chemotherapy, wherein DNA strand breaks are noted in the epithelium as well as in the cells of the submucosa, causing a relatively small proportion of cells to die quickly. However, for the majority of the remaining cells, the initial insult starts a cascade of biological events. Many of these biological events are mediated by the generation of reactive oxygen species (ROS), which occurs during and/or shortly after radiation and chemotherapy. ROS have a far-ranging and broad biological downstream impact. In addition to their ability to cause cellular injury directly, ROS effectively activate a number of central biological control mechanisms, including a specific group of transcription factors [6–9]. ROS also provokes tissue inflammation by activating macrophages and infiltrating neutrophils. Therefore, adequate reduction of ROS is essential for avoiding some of the unfortunate side effects of radiation therapy.

Hangeshashinto (HST), a Japanese traditional (Kampo) medicine, has been used as an ethical medicine for the treatment of OM, acute or chronic gastrointestinal catarrh, fermentative diarrhea, dyspepsia, gastroptosis, nervous gastritis, gastrasthenia, hang-over, belching, heartburn, stomatitis, and neurosis. HST is composed of seven crude drugs: Pinelliae Tuber (hange), Scutellariae Radix (ogon), Zingiberis Siccatum Rhizoma (kankyo), Glycyrrhizae Radix (kanzo), Zizyphi Fructus (taiso), Ginseng Radix (ninjin) and Coptidis Rhizoma (oren), as listed in Table 1. A recent clinical report has described the remedial effect of HST for chemo-radiation–induced OM [10]. Furthermore, the protective effect of HST for chemotherapy-induced OM ulcers has been tested clinically, and HST was determined to improve the grade and reduce the duration of symptoms [11, 12]. In addition, HST was demonstrated to inhibit prostaglandin E_2_ (PGE_2_) production from human oral keratinocytes stimulated with interleukin (IL)-1β [13], and to suppress PGE_2_ production, IL-6 and IL-8 in lipopolysaccharide-treated human gingival fibroblasts [14]. However, the detailed mechanisms by which HST mitigates radiation-induced mucosal damage have yet to be clearly established.

**Table 1.** Seven constituent crude drugs and 15 ingredients of hangeshashinto

Crude Drug	Amount (g)	Major ingredients
Pinelliae Tuber (hange)	5.0	corymboside
Scutellariae Radix (ogon)	2.5	baicalin, baicalein, wogonin, acteoside
Zingiberis Siccatum Rhizoma (kankyo)	2.5	[6]-shogaol, [6]-gingerol
Glycyrrhizae Radix (kanzo)	2.5	liquiritin, glycyrrhizin, glycyrrhetin
Zizyphi Fructus (taiso)	2.5	cyclic AMP
Ginseng Radix (ninjin)	2.5	ginsenoside Rg1, ginsenoside Rb1
Coptidis Rhizoma (oren)	1.0	berberine chloride, coptisine

4.5 g of dried extract of the above mixed constituents was contained in 7.5 g of hangeshashinto formulation. The crude drug names given in parenthesis are the conventional Kampo names used in Japan. The underlined ingredients are water soluble.

The antioxidative functions of HST are typically discussed from the viewpoint of its chemical activity in relation to ROS and/or other free radical species [15, 16]. Free radical–scavenging activities of HST and some of its crude drugs have also been reported. Kaneko *et al.* reported that HST suppresses nitric oxide (NO•) production in mouse macrophage–like cells [17]. Phytocomplexes obtained from the leaves of *Glycyrrihiza glabra* L. (the source of one of the constituent crude drugs of HST) can reduce 1,1-diphenyl-2-picrylhydrazyl (DPPH) radicals [18]. Glycyrrhizin, which is contained in kanzo, demonstrated a moderate suppression of superoxide (O_2_^•−^), but little or no effect on DPPH radicals [19]. Ginsenoside Rb1, one of the ingredients in ninjin, was found to directly eliminate the hydroxyl radical (•OH) [20]. Ginseng extract was declared to scavenge •OH [21]. In addition, antioxidative activity of ginsenoside (through the activation of Nrf2) has been reported [22].

In the present study, we have attempted to clarify the antioxidative and/or radiopreventive mechanisms of HST and to verify its application in relieving the effects of free radical damage resulting from radiation, one of the causes of OM.

## MATERIALS AND METHODS

### Hangeshashinto: seven constituent crude drugs and 15 ingredients

The dry powdered extracts of HST and the seven constituent crude drugs investigated in the present study were supplied by Tsumura & Co. (Tokyo, Japan). Some of the significant ingredients contained in each crude drug listed in Table 1 were also isolated and supplied by Tsumura & Co. The water-soluble ingredients underlined in the table were tested in this study.

### Chemicals

5,5-dimethyl-1-pyrroline-*N*-oxide (DMPO) was purchased from LABOTEC Co. (Tokyo, Japan). 4-hydroxyl-2,2,6,6-tetramethylpiperidine-*N*-oxyl (TEMPOL) was purchased from Sigma–Aldrich (St Louis, MO, USA). Reduced glutathione (GSH) was purchased from Wako Pure Chemical Industries (Tokyo, Japan). Other chemicals were of analytical grade. For the basic solvent of the reaction mixtures, 100-mM phosphate buffer (pH 7.0) containing 0.05-mM diethylenetriaminepentaacetic acid (DTPA), denoted hereafter as ‘PB’, was prepared and used for all experiments. Deionized water (Milli-Q system, Merck Millipore, Billerica, MA) was used for preparing the PB.

### Comparison of DMPO-OH suppression by HST for several •OH sources

When X-ray irradiation was used as an •OH source, reaction mixtures containing 30-mM DMPO and various concentrations (%w/v) of HST (0.5%, 1.0% and 2.0%) suspended in PB were prepared. The reaction mixtures were irradiated with 32 Gy X-rays (PANTAK 320S; Shimadzu, Kyoto, Japan). The dose 32 Gy was selected by taking into account the balance between the electron paramagnetic resonance (EPR) signal strength of the DMPO-OH produced and the total irradiation time (in our previous paper [23]). The conditions of X-ray irradiation are described below. The concentration of DMPO-OH generated in the reaction mixture was measured by an X-band EPR spectrometer (JES-RE1X; JEOL, Tokyo, Japan) 5 min after irradiation.

When UV irradiation of H_2_O_2_ (UV + H_2_O_2_ system) was used as an •OH source, reaction mixtures containing 30-mM DMPO, 1-mM H_2_O_2_ and various concentrations (%w/v) of HST (0.25%, 0.5%, 1.0% and 2.0%) suspended in PB were irradiated by UV light. The time course of DMPO-OH generation in the reaction mixture during UV irradiation was measured by X-band EPR for 60 min.

When the Fenton reaction system was used as an •OH source, FeSO_4_ solution was added as the starter to the reaction mixture containing DMPO and H_2_O_2_ in order to obtain final concentrations of 30-mM DMPO, 1-mM FeSO_4_ and 1-mM H_2_O_2_. The EPR signal of DMPO-OH generated in the reaction mixture was measured with X-band EPR 5 min after starting the reaction.

### X-ray irradiation

X-ray irradiation was performed using an effective energy of 80 keV under the following conditions: X-ray tube voltage of 200 kV and tube current of 20 mA, where the thickness and materials of the pre-filter were 0.5 mm copper and 0.5 mm aluminum. The dose rate of the X-ray irradiation was 3.3 Gy/min for a 300-mm distance between the X-ray tube and the sample.

### X-band EPR measurement

An aliquot (120–130 μl) of the reaction mixture was taken in a sample tube, set in a transverse electric (TE)-mode cavity using a special cell holder, and measured as soon as possible. The EPR conditions were as follows: microwave frequency of 9.4 GHz, microwave power of 4 mW, center field of 334 mT, sweep width of 10 mT, sweep speed of 5 mT/min, modulation frequency of 100 kHz, modulation amplitude of 0.079 mT and time constant of 0.03 s.

### O_2_^•−^-elimination ability of HST and constitutive crude drug extracts of HST

The temperature- and GSH-dependent reduction of TEMPOL can serve as an index of O_2_^•−^ generation in the reaction mixture [24, 25]. Reaction mixtures containing 0.1-mM TEMPOL and 1-mM GSH were prepared using PB. The reaction mixture during testing was kept in a screw-top vial and incubated in a water bath at 44°C. The time course of the EPR signal of TEMPOL in the reaction mixture was measured by X-band EPR. The centerline of the triplet EPR signal was measured. Equivalent experiments were conducted after adding 1.6-U/ml superoxide dismutase (SOD) to the reaction mixture or alternatively by bubbling N_2_ gas through the reaction mixture. Next, the experiment of adding 2.0% HST to the reaction mixture was performed, both with and without GSH. In addition, experiments adding 0.1%, 0.5% or 1.0% of HST or a crude drug extract were performed similarly.

### Simultaneous assessment of •OH-elimination ability and reducing ability of HST, constituent crude drug extracts, and ingredients contained in HST

The •OH-scavenging ability and reducing ability of HST, crude drug extracts, and the ingredients contained in HST were assessed simultaneously according to the procedure employed in a previous paper [23]. The intrinsic extent of DMPO-OH generation in the sample solution (comprised of 15-mM DMPO prepared with PB during 32 Gy X-ray irradiation), C_0int_, was previously estimated by simulation with disregard for the reduction of DMPO-OH over the course of irradiation. A reaction mixture containing 15-mM DMPO and an arbitrary concentration of a sample compound was prepared with PB. The reaction mixture containing the sample compound was irradiated with 32 Gy X-rays, and the reduction rate of DMPO-OH in the reaction mixture with the sample compound, k_exp_, was obtained from the decay curve of DMPO-OH after X-ray irradiation. The concentration of DMPO-OH at the end of X-ray irradiation, C_0exp_, was estimated by extrapolating the decay curve to the time at which X-ray irradiation ceased. Then, the expected concentration of DMPO-OH in the reaction mixture at the end of X-ray irradiation, C_0net_, was calculated using C_0int_ and k_exp_. The percentage difference of C_0exp_ compared with C_0net_ was estimated as the percentage of •OH elimination, i.e., (1−C_0exp_/C_0net_) × 100. The reduction rate of DMPO-OH, k_exp_ and the percentage of •OH elimination were estimated for each sample concentration. Reduction rates of DMPO-OH, k_exp_, in the samples measured were plotted versus the concentration of the subject compound, and then the second order rate constant, k_2nd_, was obtained from the slope of the plot. The concentration dependence of the percentage of •OH elimination was similarly obtained, and then the half-maximal inhibitory concentration (IC_50_) value was estimated. X-ray irradiation and the subsequent EPR measurements were repeated three times for each of the sample solution concentrations. For the spin-trapping experiment, the second peak from the lower field of four lines of the DMPO-OH EPR spectrum was recorded under the following conditions: microwave frequency 9.45 GHz, microwave power 2 mW, lower magnetic field 336.1 mT, sweep width 1.25 mT, sweep speed 5 mT/min (15 s for 1.25 mT), modulation frequency 100 kHz, modulation amplitude 0.063 mT and time constant 0.03 s.

### Statistical test

The statistical differences were estimated with alternative Student's or Welch's *t*-test. The suitable test for the data was automatically selected according to variance of the data. Grades of significance were estimated by *P* < 0.05, *P* < 0.01 and *P* < 0.001.

## RESULTS AND DISCUSSION

**Fig. 1.**
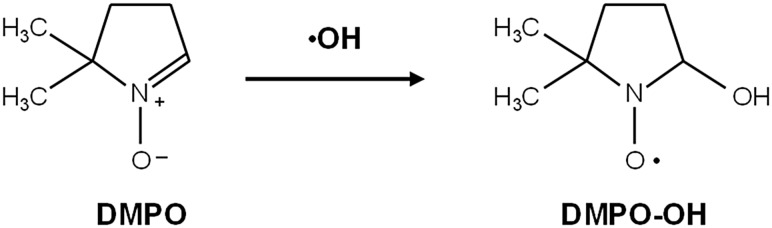
Spin trapping of •OH by DMPO. Diamagnetic DMPO reacts with •OH and produces paramagnetic DMPO-OH, which is a relatively stable nitroxyl radical. DMPO-OH provides a characteristic 1:2:2:1 four-line EPR signal.

**Fig. 2.**
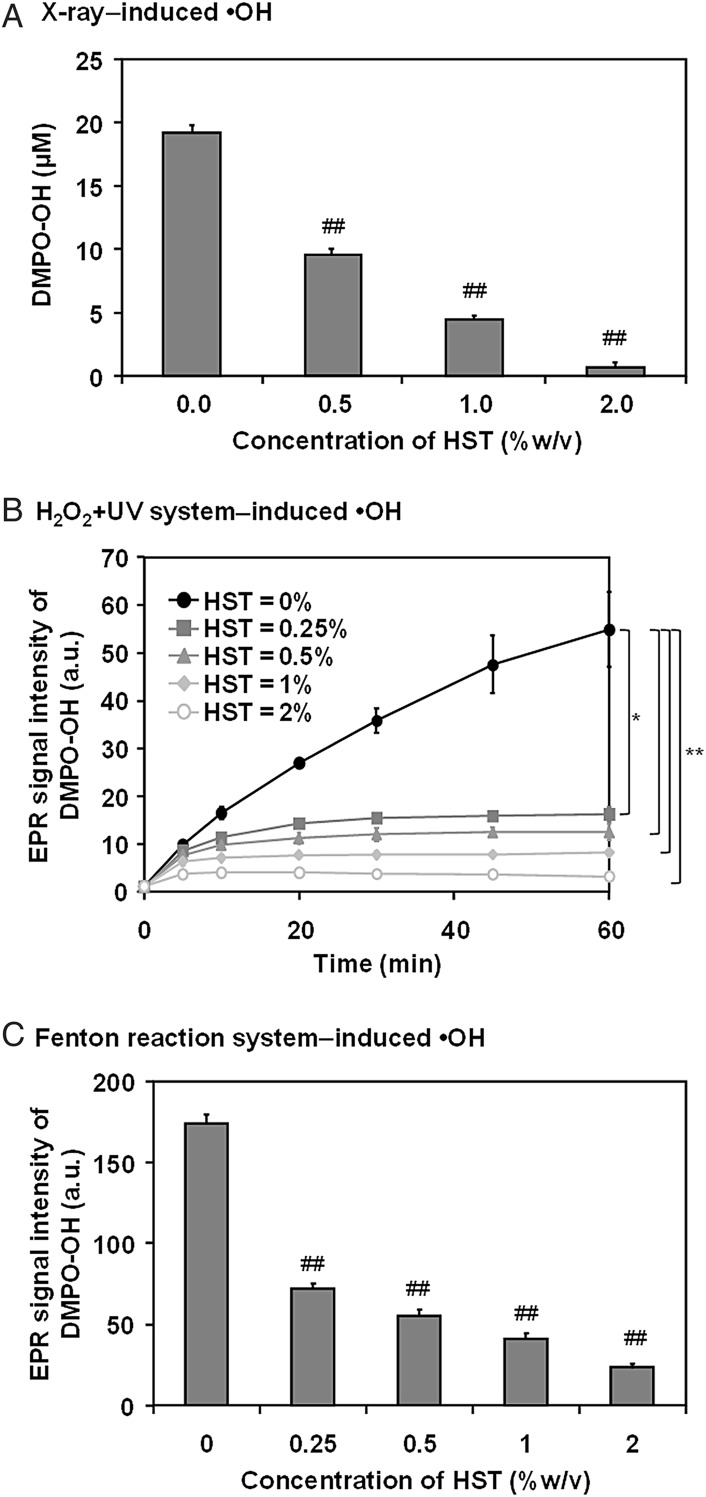
Suppression of DMPO-OH generation by hangeshashinto (HST) for several •OH sources. (**A**) Suppression of X-ray–induced DMPO-OH by HST. Reaction mixtures containing 30-mM DMPO and various concentrations of HST were irradiated with 32 Gy X-rays. X-band EPR measurements were conducted 5 min after X-ray irradiation. (**B**) Suppression of UV + H_2_O_2_ induced DMPO-OH by HST. Reaction mixtures containing 30-mM DMPO, 1-mM H_2_O_2_ and various concentrations of HST were irradiated with UV light. The time course of DMPO-OH generation during UV irradiation was measured. (**C**) Suppression of the Fenton reaction–induced DMPO-OH by HST. Reaction mixtures containing 30-mM DMPO, 1-mM H_2_O_2_, 1-mM FeSO_4_ and various concentrations of HST were prepared and measured by X-band EPR. The columns/marks and error bars indicate the mean ± standard deviation based on three samples. *, ** and ^##^ indicates significance compared with the control as *P* < 0.05, *P* < 0.01 and *P* < 0.001, respectively.

The spin-trapping agent, DMPO, is able to scavenge •OH and provides a characteristic four-line EPR spectrum of the •OH spin adduct DMPO-OH (as shown in Fig. 1) in the reaction mixture with an •OH-source. The spin adduct, DMPO-OH, is a relatively stable free radical, which can be measured by EPR at room temperature. An antioxidant can compete with this spin-trapping agent and reduce the EPR signal intensity of DMPO-OH. The •OH-scavenging ability can then be estimated by the decrement of the EPR signal intensity. Figure 2 shows a comparison of the suppression of DMPO-OH generation by HST suspended in the reaction mixture along with several •OH sources. Figure 2A shows that the concentration of X-ray–induced DMPO-OH generated in the reaction mixture decreased almost linearly with increasing HST concentration. Figure 2B and C indicate suppression of DMPO-OH generation by HST suspension in both the UV + H_2_O_2_ and Fenton reaction systems, respectively. Suppression of DMPO-OH generation in the UV + H_2_O_2_ and Fenton reaction systems was found to be dependent on HST concentration, but the dependency was not linear. A relatively small concentration of HST, in this case, 0.25%, resulted in a relatively large reduction in DMPO-OH generation. This fact suggests that HST can chemically affect the chemical and/or photochemical •OH-generating reactions.

In the analysis, whether an antioxidant can affect a chemical and/or a photochemical •OH source (i.e. the Fenton reaction system and/or the UV + H_2_O_2_ system) must be considered. X-ray irradiation of an aqueous reaction mixture can generate •OH (by direct ionization of water molecules), which cannot be affected by antioxidants.

**Fig. 3.**
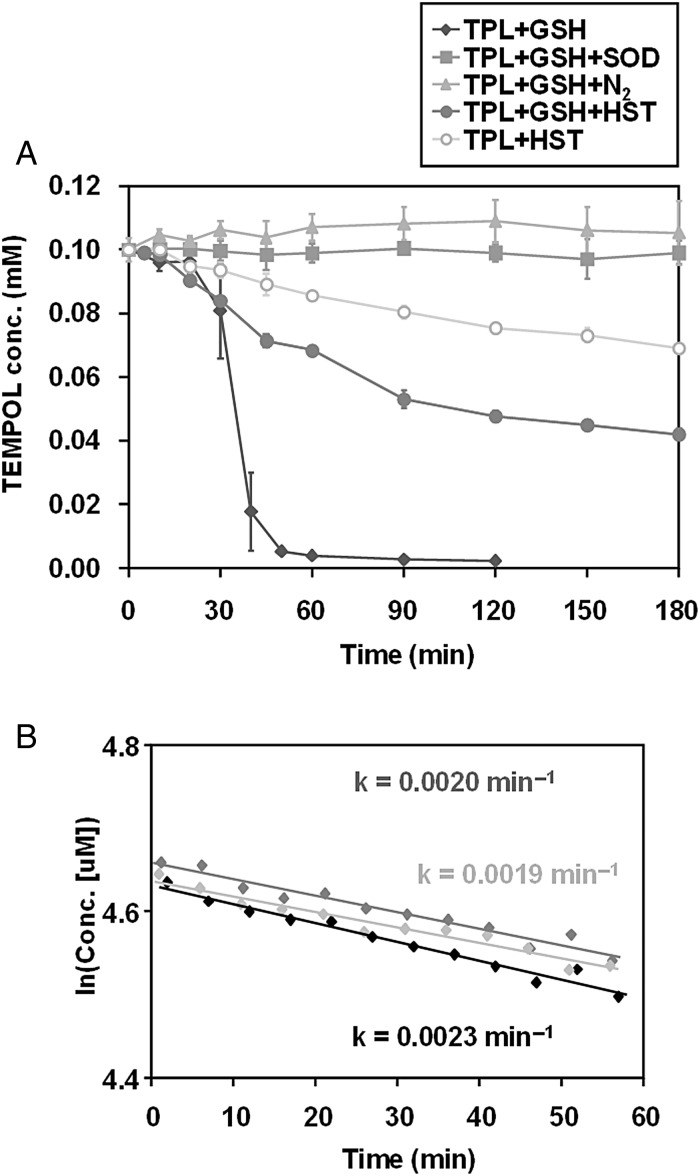
Suppression of O_2_^•−^ by HST. GSH-dependent TEMPOL reduction at 44°C was used as an index of O_2_^•−^ generation in the reaction mixture. (**A**) Comparison of the reduction profiles of TEMPOL under several experimental conditions. (**B**) Direct reduction of DMPO-OH by HST. Each mark indicated in (B) represents an independent experiment.

In Fig. 3A, HST demonstrates an O_2_^•−^-elimination ability and a stable radical–reducing ability. It has been reported that the heating of an aqueous solution containing oxygen and GSH can generate ROS, mainly as O_2_^•−^ [24, 25]. The O_2_^•−^-scavenging ability of HST was estimated using a combination of the O_2_^•−^ source and TEMPOL (as an O_2_^•−^-reactive molecular probe). As shown by the solid diamonds in Fig. 3A, upon incubating a reaction mixture containing TEMPOL and GSH at 44°C, the TEMPOL shows a steep reduction following a short time delay. This characteristic TEMPOL reduction under conditions of coexisting GSH at 44°C was halted by adding SOD to the reaction mixture (solid squares in the figure) or bubbling N_2_ gas through the reaction mixture (solid triangles). This suggests that O_2_^•−^ is related to the observed TEMPOL reduction, and that this TEMPOL reduction can be an index of O_2_^•−^ generation in the reaction mixture. After suspending 2.0%-HST in the reaction mixture, a gradual first-order decay of TEMPOL was observed, rather than the previously observed steep decay following a short time delay (solid circles in the figure). This indicates that HST eliminated O_2_^•−^ generation in the reaction mixture, and simultaneously demonstrated that HST directly reduced TEMPOL. As evidence, when 0.1-mM TEMPOL was incubated with 2.0% HST at room temperature, TEMPOL was observed to decrease gradually, as shown by the open circles in Fig. 3A. The average decay rate was determined as 0.0020 ± 0.0002 min^−1^ (Fig. 3B, *n* = 3). The value of t_1/2_ was calculated to be 5.6 h. However, the decreased EPR signal of TEMPOL with HST addition was restored by adding 2-mM K_3_Fe(CN)_6_ into the reaction mixture (data not shown), which indicates that this EPR signal decay was mainly due to direct one-electron reduction of TEMPOL. HST has a relatively strong reducing ability—like that of ascorbic acid, which can reduce stable nitroxyl free radicals directly. These facts suggest that HST can function as an antioxidant by both scavenging ROS and reducing oxidized biomolecules.

This relatively strong reducing ability of HST can of course reduce DMPO-OH, which is also a nitroxyl radical, less stable than TEMPOL. This fact suggests that the results of the suppression of X-ray–induced DMPO-OH generation (shown in Fig. 2A) is not only due to •OH scavenging but also to reducing DMPO-OH. In fact, the original decay rate of X-ray–induced DMPO-OH generated in the simple reaction mixture (prepared with only PB and 15-mM DMPO) (i.e. 0.0193 ± 0.0016 min^−1^, for *n* = 12, t_1/2_ = 36.1 min) increased when 2.0% HST was added to the reaction mixture after X-ray irradiation. The decay rate of DMPO-OH with coexisting 2.0% HST was 0.0980 ± 0.0091 min^−1^ (*n* = 3), which yielded a t_1/2_ value of 7.1 min. It is important to remember that some of the antioxidants can reduce the spin adduct to an EPR silent form.

Because HST is a formulation containing an assortment of seven crude drugs, the main antioxidative behaviors—O_2_^•−^-elimination abilities, •OH-elimination abilities and reducing abilities of crude drug extracts—are measured individually.

At first, the O_2_^•−^-elimination ability was estimated using the GSH-dependent TEMPOL reduction reaction system as an index of O_2_^•−^ generation [24, 25]. Heating a reaction mixture containing GSH and TEMPOL resulted in an EPR signal loss for the TEMPOL. This TEMPOL reduction demonstrated a characteristic decay profile, which consists of a delay prior to a steep reduction. This reaction can be employed as an index of O_2_^•−^ generation, because the reaction was suppressed by SOD or by deoxygenating the reaction mixture.

**Fig. 4.**
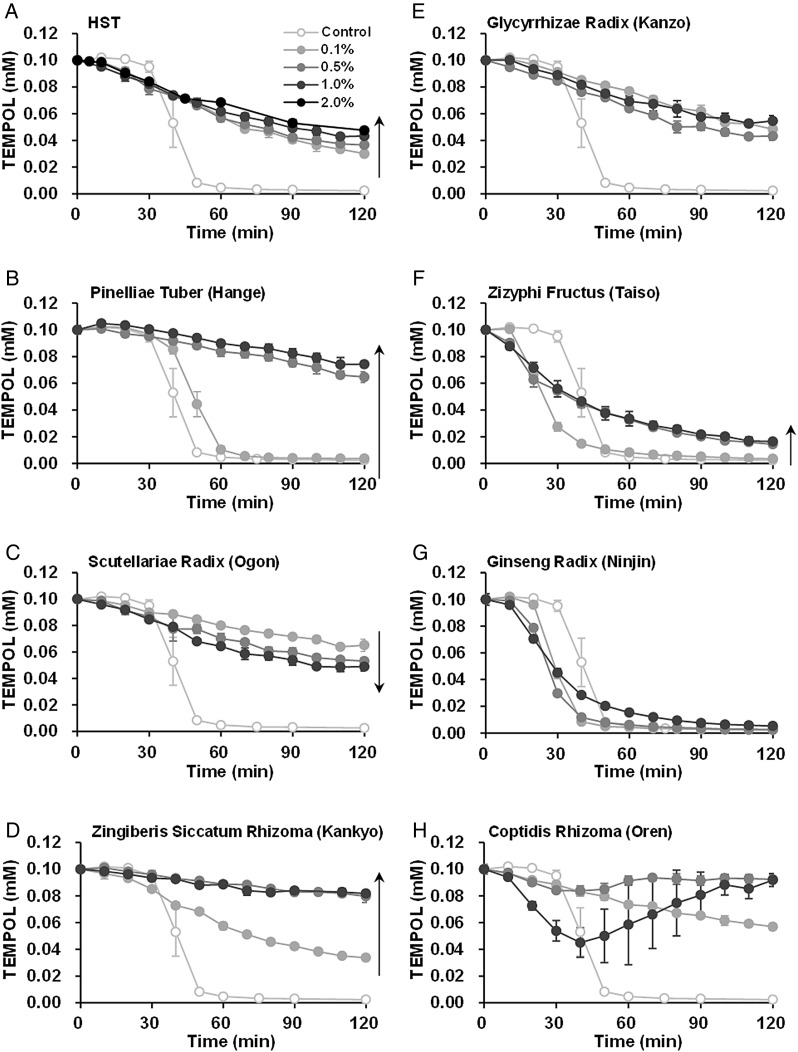
Comparison of O_2_^•−^ elimination/suppression of the constituent crude drug extracts of HST: (**A**) HST, (**B**) hange, (**C**) ogon, (**D**) kankyo, (**E**) kanzo, (**F**) taiso, (**G**) ninjin and (**H**) oren. GSH-dependent TEMPOL reduction at 44°C was used as an index of O_2_^•−^ generation in the reaction mixture.

Figure 4 shows a comparison of the O_2_^•−^-elimination abilities of HST and that of the seven crude drug extracts. Figure 4A shows reaction profiles of the GSH-dependent TEMPOL reduction for a range of doses of HST. As described above, the reaction mixtures with added HST exhibited profiles indicative of first-order decay, i.e. the results of simultaneous O_2_^•−^ elimination and direct reduction of TEMPOL rather than the steep TEMPOL decay shown in the control reaction mixture. The reduction of TEMPOL was somewhat suppressed (dose dependently) by HST, rather than accelerated. This result suggests that O_2_^•−^ elimination was advantageous for 0.1–2.0% HST in comparison with the direct reduction of TEMPOL. The reaction profiles of GSH-dependent TEMPOL reduction for each crude drug extract are shown in Fig. 4B–H. Hange (Fig. 4B), ogon (Fig. 4C), kankyo (Fig. 4D) and kanzo (Fig. 4E) demonstrated suppression with steep TEMPOL decay, which indicates O_2_^•−^ elimination and/or O_2_^•−^ suppression. Because hange (Fig. 4B) and kankyo (Fig. 4D) suppressed TEMPOL reduction in a dose-dependent manner, hange and kankyo exhibit a predominantly O_2_^•−^-eliminating rather than reducing ability. Ogon, however, demonstrated a dose-dependent acceleration of TEMPOL reduction (Fig. 4C). Therefore, ogon exhibits a predominantly reducing ability rather than O_2_^•−^-eliminating ability. Kanzo demonstrated nearly equivalent reduction profiles for the 0.1–1.0% doses (Fig. 4E). Therefore, it may be that in this case the O_2_^•−^ elimination and reduction abilities function equally. Taiso demonstrated no suppression of the steep TEMPOL decay at a 0.1% concentration, but rather made the TEMPOL reduction delay shorter relative to that of the control (Fig. 4F). Because the shapes of the reduction profiles of TEMPOL with taiso addition changed to first-order decay, like that at a 0.5% or 1.0% taiso concentration, taiso may have a very weak O_2_^•−^-elimination ability (Fig. 4F). Ninjin also demonstrated a shortened time delay for the steep TEMPOL decay; however, the profile shapes did not change at higher concentrations (Fig. 4G). The reaction mixture containing 0.1% oren demonstrated a moderate first-order decay-like profile (Fig. 4H). When the reaction mixture contained a 0.5% or 1.0% oren concentration, the TEMPOL signal temporarily decreased and then recovered. Oren demonstrated re-oxidation of TEMPOL at higher concentrations. The slopes of reduction of TEMPOL by oren, which can be observed 0–30 min after starting the reaction, however, look steeper compared with those of ogon (Fig. 4C) at corresponding concentrations. This fact suggests that the intrinsic reduction ability of oren may be stronger than that of ogon. Oren has relatively strong reducing ability and moderate oxidative ability.

**Fig. 5.**
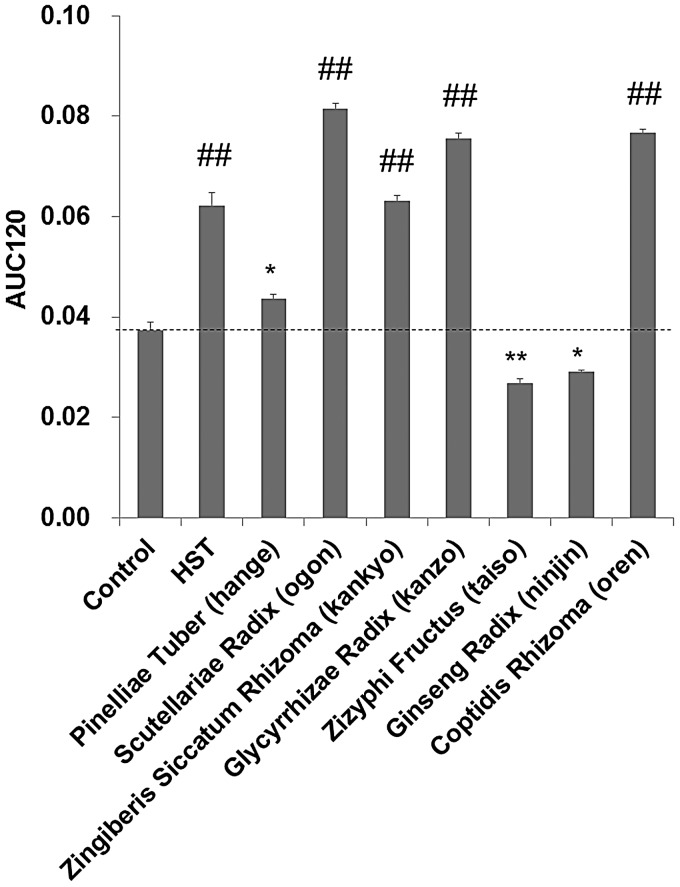
Comparison of O_2_^•−^ elimination/suppression of the constituent crude drug extracts of HST using the areas under the curves estimated from the reaction profiles with the 0.1% sample contents given in Fig. 4 for the time window 0–120 min (AUC120). *, ** and ^##^ indicates significance compared with the control as *P* < 0.05, *P* < 0.01 and *P* < 0.001, respectively.

The areas under the curves (AUCs) estimated from the reaction profiles given in Fig. 4 for the time window 0–120 min (AUC120) were obtained for the reaction profiles for 0.1% HST and for each crude drug extract. A dose of HST in the clinical treatment is 2.5–3.75 g, which may be swallowed down with a glass of water (200 ml). The HST is diluted by the water to 1.25–1.9%. In the gastrointestinal tract, HST may be further diluted to ≤0.1%. Figure 5 shows a comparison of the AUC120 data. AUC120 values higher than the control may be indicative of O_2_^•−^ elimination and/or O_2_^•−^ suppression. Based on the AUC120 data, HST, hange, ogon, kankyo, kanzo and oren demonstrated O_2_^•−^-eliminating and/or O_2_^•−^-suppressing abilities.

**Fig. 6.**
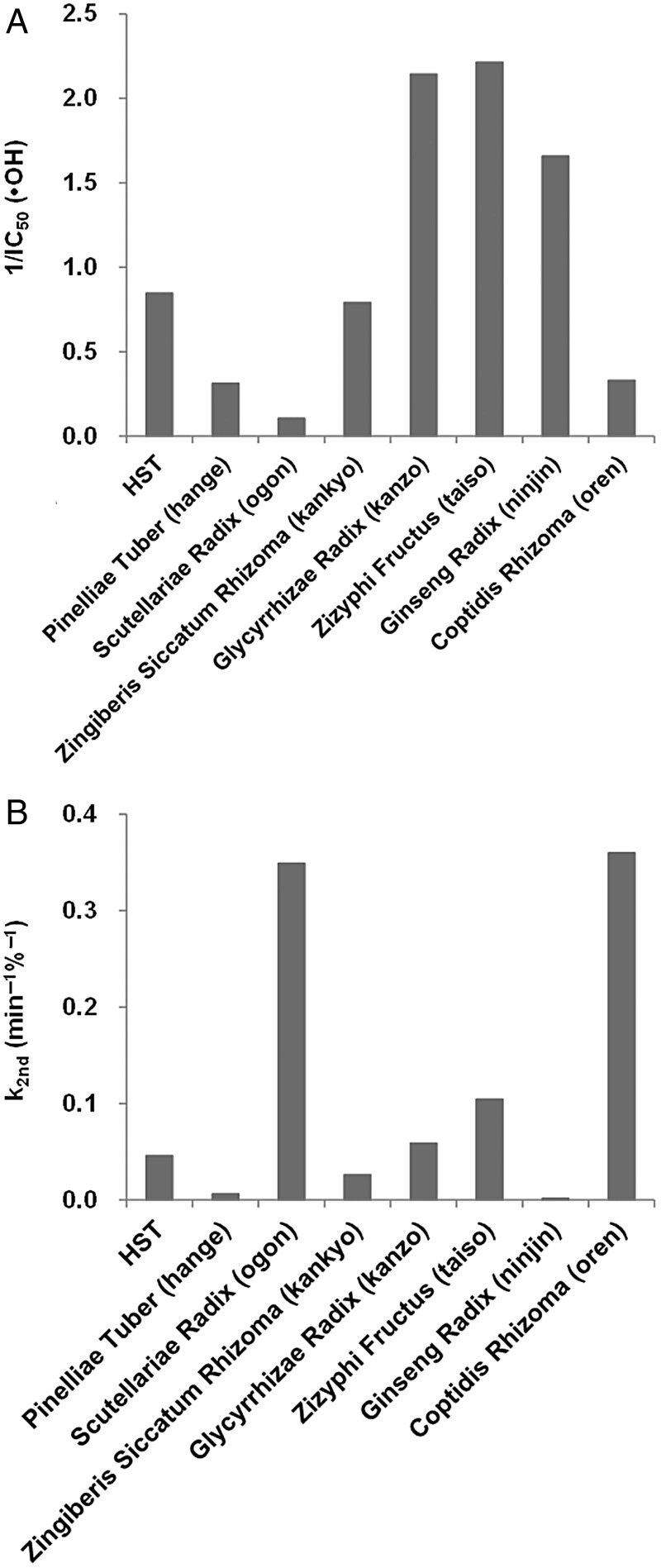
Suppression of X-ray–induced DMPO-OH by the constituent crude drug extracts of HST. Reaction mixtures containing 30-mM DMPO and various concentrations of the crude drug extracts were irradiated by 32 Gy X-rays. (**A**) •OH-elimination ability, IC_50_, and (**B**) reducing ability, k_2nd_, were estimated by an analysis of the time course of the EPR spectrum.

Next, the •OH-eliminating abilities and reducing abilities of the individual crude drugs were assessed using a modified application of the EPR spin-trapping technique [23]. This is a new method for the simultaneous assessment of the •OH-eliminating ability and the reducing ability of water-soluble antioxidants. The method has the advantage that the •OH-eliminating ability of an antioxidant can be estimated, even if the antioxidant has a relatively strong reducing ability. Using this method, the •OH-eliminating ability, IC_50_, and the reducing ability, k_2nd_, was estimated for each crude drug extract. Figure 6 shows a comparison of the •OH-eliminating abilities (Fig. 6A) and the reducing abilities (Fig. 6B) of the crude drug extracts of HST. Lower IC_50_ values indicate higher levels of •OH elimination activity. To present the data easily and visually, the reciprocals of the IC_50_ values of •OH elimination are indicated as a bar graph in Fig. 6A. Kanzo, taiso and ninjin demonstrated relatively high •OH-elimination abilities. These three crude drugs contain relatively high concentrations of sugars. It has been reported that sugars have relatively high •OH-elimination abilities [23]. The higher •OH-elimination abilities of these crude drugs are probably imparted by the sugar contents. Figure 6B shows a comparison of the reducing abilities of the crude drugs. Here, higher k_2nd_ values indicate higher reducing abilities. Ogon and oren demonstrate relatively strong reducing abilities.

Finally, to clarify which ingredients of HST provide the principal antioxidative effects, the •OH-elimination ability (IC_50_) and reducing ability (k_2nd_) of the water-soluble ingredients were estimated. The results are summarized in Table 2. The •OH-elimination abilities of the ingredients tested were demonstrated reasonably well in this study. Glycyrrhizin, which is an ingredient of kanzo, demonstrated a markedly high •OH-eliminating ability (i.e. a lower IC_50_ value) relative to the other ingredients tested. Glycyrrhetin, which is a simple aglycone of glycyrrhizin, demonstrated a low •OH-eliminating ability relative to glycyrrhizin; nonetheless, this ability was still as good as that of DMSO. DMSO and mannitol, which are commonly employed •OH scavengers in biological experiments, yielded IC_50_ values of 6.9 mM and 15.6 mM, as given in a previous report [23]. The ingredients of HST tested in the present study exhibited similar or higher •OH-elimination abilities relative to those of common •OH scavengers, except for ginsenoside Rb1, which exhibited a relatively moderate •OH-elimination ability.

**Table 2.** •OH-elimination abilities and reducing abilities of ingredients of hangeshashinto

Ingredient	Crude drug	M.W.	Reducing ability	IC_50_ of •OH elimination
			k_2nd_ (min^−1^M^−1^)	(mM)
acteoside	ogon	624.6	0.203	5.32
glycyrrhizin	kanzo	822.9	n.d.	0.53
glycyrrhetin	kanzo	470.7	n.d.	6.63
cyclic AMP	taiso	329.2	n.d.	6.64
ginsenoside Rg1	ninjin	801.2	n.d.	3.68
ginsenoside Rb1	ninjin	1109.3	n.d.	42.61
berberine chloride	oren	371.8	0.455	6.17
DMSO	-----	78.1	n.d.	6.9^a^
SDS	-----	288.7	n.d.	46.0^a^

M.W. = molecular weight, n.d. = not detected. ^a^Values are referenced from Ueno *et al.* [23].

Because •OH has a very high reactivity, •OH reacts with nearly anything. Hence, an elongated molecule, such as sugar, has a great potential for encountering •OH; therefore, the •OH-eliminating ability of an elongated molecule should be higher than that of a compact molecule. In addition, a simple carbon chain, such as sodium dodecyl sulfate (SDS), has a moderate •OH-eliminating ability. SDS has a larger molecular weight than mannitol; however, it exhibited a lower •OH-eliminating ability than mannitol. The IC_50_ value of SDS reported in a previous work was 46.0 mM [23]. Therefore, the ring structure, double bond, ether bond and/or ketone moiety in the molecule may be important for reactivity with •OH.

Stable radical–reducing abilities were demonstrated only by acteoside (an ingredient of ogon) and berberine chloride (an ingredient of oren) out of all the chemical ingredients tested in these experiments. The k_2nd_ values of acteoside and berberine chloride were 0.203 and 0.455 min^−1^M^−1^, respectively. These k_2nd_ values are markedly lower than that of ascorbic acid, which yields a k_2nd_ value of 4986 min^−1^M^−1^. Therefore, the relatively strong reducing ability of oren and ogon observed in Fig. 6B may be the result of a total effect created in coordination with other reducing contents in these crude drugs. The reducing ability of the ogon extract was verified by both EPR spin-probing and spin-trapping experiments. In addition, the reducing ability of acteoside, an ingredient of ogon, was observed in the spin-trapping experiment. On the other hand, the reducing ability of the oren extract and berberine chloride, an ingredient of oren, was observed in the EPR spin-trapping experiments, whereas a marked reducing ability of the oren extract could not be observed in the EPR spin-probing experiment at higher concentrations (Fig. 4H).

The oren extract demonstrated its role as an oxidant rather than as a reductant in the EPR spin-probing experiment at higher concentrations (Fig. 4H). This competing oxidative activity of oren resulted in the apparently weak reducing activity on TEMPOL at the higher concentrations. The oren extract was able to reduce nitroxyl radicals, i.e. DMPO-OH and TEMPOL. TEMPOL was mainly one-electron–reduced to its hydroxylamine form, which can be easily reoxidized to the nitroxyl radical form; however, a relatively unstable DMPO-OH molecule may be further reduced to unknown decomposition products, which cannot be restored to DMPO-OH. The role of oren as an oxidant may function to maintain the catalytic total redox action of the HST.

In this study, the antioxidative abilities of HST and of the other crude drugs were tested as suspensions in aqueous buffer solutions. In addition, the water-soluble ingredients only of HST were tested in this study. The lipophilic contents of HST may also serve various biological roles, taking into account the fact that HST is operative on the internal surface mucosa of the oral and/or intestinal cavity.

Three important ROS associated with radiobiological effects are •OH, O_2_^•−^ and H_2_O_2_. The •OH is one of the initial products of water radiolysis. Because •OH can serve as a source of the other ROS, the primary regulation of •OH using antioxidants may be vital for radioprotection. Generation of O_2_^•−^ and/or H_2_O_2_ is accompanied by some O_2_ consumption. H_2_O_2_ is a relatively stable molecule compared with •OH and O_2_^•−^. H_2_O_2_ can, therefore, move a relatively long distance and has the potential for accumulation. A reaction of H_2_O_2_ and O_2_^•−^ can also yield •OH. In addition, H_2_O_2_ can react with biological metal ions such as Cu^+^ and/or Fe^++^ and subsequently form •OH. Some functional molecules may be oxidatively injured by •OH. Such oxidized molecules can be reduced by some reductants to recover their function.

O_2_^•−^ is also generated by radiation-injured mitochondria for several days after irradiation [26, 27]. Duration of OM may be due to such a delayed O_2_^•−^ generation. Therefore, regulation of O_2_^•−^ and H_2_O_2_ is an important secondary measure for radioprotection and shortening of the duration of OM.

To regulate radiobiological effects efficiently, multiple abilities, such as •OH elimination/suppression, O_2_^•−^ elimination/suppression, H_2_O_2_ elimination/suppression, and reducing oxidized molecules are essential. The investigation of the Japanese traditional medicine, HST, conducted in the present study demonstrated that HST has multiple antioxidative abilities. The main antioxidative effect of HST is probably a combined result of ROS scavenging, a role as a reductant, and some minor oxidative activities. The multiple antioxidative abilities of HST appear to be the fundamental in providing effective protection and duration against radiation-induced mucosal and/or gastrointestinal inflammation.

## CONFLICT OF INTEREST

The authors report no conflicts of interest. Financial support for this study was provided by Tsumura & Co. Chinami Matsumoto, Yuji Omiya, Yoshio Kase and Masato Fukutake are employees of Tsumura & Co.

## FUNDING

This work was funded by Tsumura & Co. Funding to pay the Open Access publication charges for this article was provided by Tsumura & Co.
